# Density functional theory modeling of chromate adsorption onto ferrihydrite nanoparticles

**DOI:** 10.1186/s12932-018-0053-8

**Published:** 2018-03-01

**Authors:** James D. Kubicki, Nadine Kabengi, Maria Chrysochoou, Nefeli Bompoti

**Affiliations:** 1 Department of Geological Sciences, University of Texas at El Paso, El Paso, USA; 2 Department of Chemistry and Department of Geosciences, Georgia State University, Atlanta, GA USA; 3 Civil and Environmental Engineering Department, University of Connecticut, Storrs, CT USA

## Abstract

Density functional theory (DFT) calculations were performed on a model of a ferrihydrite nanoparticle interacting with chromate (${\text{CrO}}_{4}^{2 - }$) in water. Two configurations each of monodentate and bidentate adsorbed chromate as well as an outer-sphere and a dissolved bichromate (${\text{HCrO}}_{4}^{ - }$) were simulated. In addition to the 3-D periodic planewave DFT models, molecular clusters were extracted from the energy-minimized structures. Calculated interatomic distances from the periodic and cluster models compare favorably with Extended X-ray Absorption Fine Structure spectroscopy values, with larger discrepancies seen for the clusters due to over-relaxation of the model substrate. Relative potential energies were derived from the periodic models and Gibbs free energies from the cluster models. A key result is that the bidentate binuclear configuration is the lowest in potential energy in the periodic models followed by the outer-sphere complex. This result is consistent with observations of the predominance of bidentate chromate adsorption on ferrihydrite under conditions of high surface coverage (Johnston Environ Sci Technol 46:5851–5858, 2012). Cluster models were also used to perform frequency analyses for comparison with observed ATR FTIR spectra. Calculated frequencies on monodentate, bidentate binuclear, and outer-sphere complexes each have infrared (IR)-active modes consistent with experiment. Inconsistencies between the thermodynamic predictions and the IR-frequency analysis suggest that the 3-D periodic models are not capturing key components of the system that influence the adsorption equilibria under varying conditions of pH, ionic strength and electrolyte composition. Model equilibration via molecular dynamics (MD) simulations is necessary to escape metastable states created during DFT energy minimizations based on the initial classical force field MD-derived starting configurations.

**Electronic supplementary material:**

The online version of this article (10.1186/s12932-018-0053-8) contains supplementary material, which is available to authorized users.

## Introduction

Adsorption is a critical process in environmental chemistry that can control the fate and transport of aqueous species [1]. Oxyanion adsorption onto Fe-oxides and Fe-hydroxides is particularly strong in many instances as strong covalent bonds can be formed between species such as carbonate, phosphate, arsenate and chromate and phases such as hematite (α-Fe_2_O_3_), goethite (α-FeOOH) and ferrihydrite (5Fe_2_O_3_·9H_2_O). Chromate is of particular interest because it is a common and hazardous contaminant [2]. Ferrihydrite is a common adsorbing phase in the environment because it is often the first phase to precipitate from Fe-saturated aqueous solutions due to kinetic control [3]. Consequently, this study focuses on chromate-ferrihydrite interactions due to its practical importance; however, we also use this model system as a case study for comparing computational results to EXAFS, micro-calorimetry and Attenuated Total-Reflection Fourier-Transform Infrared (ATR FTIR) data. Methods tested on this system can then be applied to various other environmental chemistry problems, especially those involving oxyanion adsorption to metal oxides and hydroxides.

The most common experimental method to study environmental adsorption chemistry has been to perform adsorption isotherm experiments involving selected solid phases and varying concentrations of an adsorbent. This approach provides data on the overall affinity of a given element or compound for a particular solid; however, Villalobos and coworkers have clearly shown that the adsorption isotherm can vary significantly depending on the crystal habit of the substrate involved [4, 5]. In these papers, adsorption of species such as Pb^2+^ and chromate onto goethite was inversely proportional to goethite specific surface area—a result contrary to expectation. The reason for this inverse relationship was the change in the crystal surfaces present with certain faces such as the (010) and (210) preferentially serving as excellent adsorption substrates. The fundamental chemical reason for this behavior is the higher concentration of the more reactive Fe-OH (OH bonded to a single Fe atom) moieties on the (010) and (210) surfaces, compared to less reactive Fe_2_OH and Fe_3_OH (OH bonded to a two or three Fe atoms, respectively) moieties that are more dominant on other surfaces such as (100) and (001). There is evidence showing that the observed point-of-zero charge on Al_2_O_3_ varies with crystal habit [6], and this observation can be attributed to a similar phenomenon, with different pK_a_ values of various Al_n_OH sites being present on surfaces. One can conclude that similar surface chemistry is involved in prior studies that observed changes in adsorption amounts and mechanisms onto hematite as a function of crystal habit [7]. A similar effect may be present in a study that observed changes in relative fractions of adsorbing species of chromate, selenite, and sulfate onto ferrihydrite as a function of Al-substitution [8]. In this case, Al may change the habit of the solid as well as changing the pK_a_s of the surface metal-OH groups [9]. Hence, in order to understand adsorption reactions relevant to environmental chemistry, it is necessary to model these reactions on all possible adsorbing surfaces [10].

Molecular modeling of mineral–water interfaces and adsorption reactions have predominantly been performed on 3-D periodic ideal crystal surfaces and/or with truncated molecular clusters [11]. Although these methods have supplied useful information and reasonable agreement with observed spectroscopic properties [12], the former do not include defects such as step edges and corners ubiquitous on naturally-formed surfaces, and the latter allow for over-relaxation of the solid substrate and do not include longer-range effects. Hummer et al. [13, 14], concluded that these edges and corners can contribute significantly to a nanoparticle’s overall surface energy, in addition to being more reactive towards adsorbing species. Because ferrihydrite occurs as nanoparticles (it has been designated a nano-mineral [3]), it is necessary to model chromate adsorption onto ferrihydrite including these surface defects. We have done this in this study by modeling a ferrihydrite nanoparticle interacting with chromate in water under 3-D periodic boundary conditions.

Another issue addressed in this current research is the supposition of a single adsorption mechanism associated with a given sorbent-sorbate pair under a given set of conditions (i.e., pH, concentration, temperature, etc.). For example, studies have concluded that a single species exists for phosphate-goethite adsorption at a given pH and that the predominant species changes as pH changes [15, 16]. However, other results show that several species under any given set of experimental conditions were required in order to explain all of the observed ATR FTIR peaks of phosphate adsorbed onto goethites [10]. Specifically for chromate adsorption on iron oxides, the authors have performed several studies on ferrihydrite using complementary techniques (ATR FTIR, EXAFS, micro-calorimetry) across a wide variety of experimental conditions [8, 17, 18]. Collectively, these studies have shown that three possible species are present on the surface (binuclear bidentate, monodentate and outer-sphere) and the relative proportion of these is highly dependent on parameters such as the pH, surface coverage, ionic strength and presence of Al in the structure. In general, lower pH and higher surface coverage tend to favor bidentate complexes, whereas monodentate forms when the surface coverage is low, either due to the presence of insufficient positive charge on the surface (high pH) or low sorbent concentration. Outer-sphere complexation is favored by the presence of Al impurities within the crystal and was found to be less than 5% in pure Fe-ferrihydrite [17]. The outstanding question in terms of molecular modeling is how accurately these observations can be represented by DFT calculations.

The hypothesis of this study is that DFT geometry optimizations and frequency analyses will result in a model consistent with the EXAFS, IR, and calorimetry data under a specific set of experimental conditions. In order to discover which model best reproduces these experimental observables, a combination of periodic models, which can better represent the adsorption reaction for comparison with calorimetry, and cluster models, where analytical frequencies and IR intensities can be calculated, were used. The cluster models were derived from the periodic models in order to perform self-consistent comparisons of the relative adsorption energies and the IR frequencies of the models with the data. In addition, use of the nanoparticle in the calculations allows for investigation of adsorption at different site types on the model ferrihydrite in contrast with most DFT studies of adsorption where periodic surfaces are used. This step is necessary to test a second hypothesis that site variability on surfaces, especially those of nanoparticles, strongly influences adsorption energies. This leads to difficulty in interpreting calorimetric data with regard to a single type of surface complex, because at a given concentration, the observed ΔH_ads_ will be an average of all types of sites present. A third hypothesis examined is that adsorption of oxyanions may occur as two or more species in equilibrium as has been suggested previously [8, 10, 19, 20]. When these latter two hypotheses are correct, then the practice of fitting adsorption isotherms with a single surface complex is unrealistic and needs to be replaced with more complex models that account for this variability.

## Methods

### Model construction

**Fig. 1:**
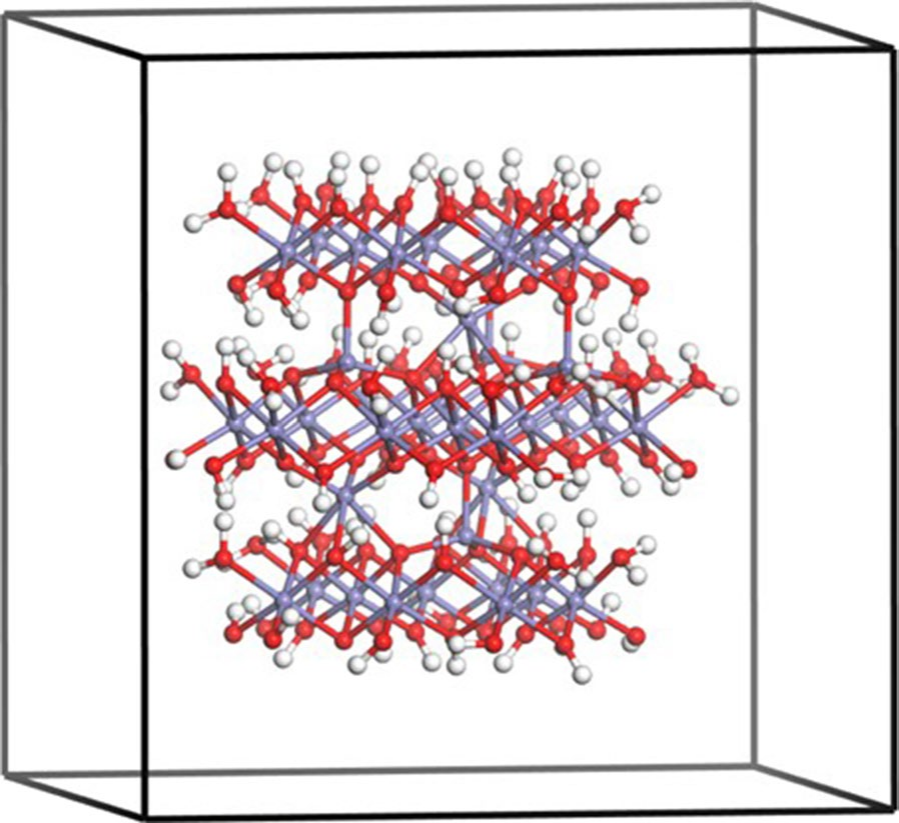
1.6 nm particle, Fe_38_O_112_H_110_ based on the structure of Michel et al. [21] as refined by Pinney et al. [23] and surface construction of Hiemstra [24] inside 20 × 20 × 20 Å^3^ 3-D periodic cell. H = white, O = red, Fe = blue-gray

**Fig. 2:**
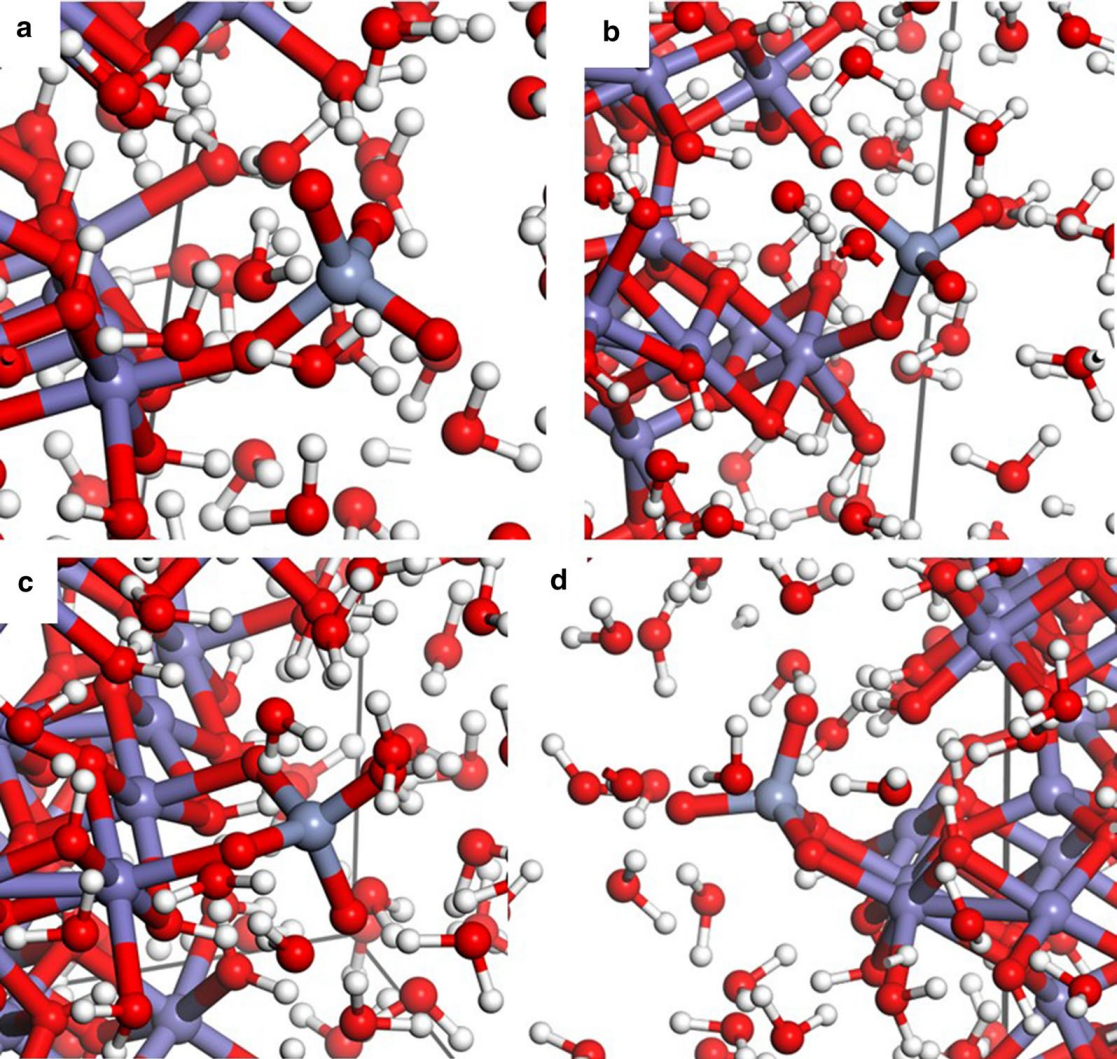
The four inner-sphere configurations (**a** = Mono(A), **b** = Mono(B), **c** = Bi(A) and **d** = Bi(B)) were constructed to test the thermodynamic favorability of surface site types. H = white, O = red, Fe = violet-gray, Cr = magenta

A charge-neutral ferrihydrite nanoparticle model (Fe_38_O_112_H_110_) was built upon the experimentally-determined structure [21], and periodic structure as calculated by two research groups [22, 23]. A central tetrahedrally-coordinated Fe atom was selected in the Visualizer module of Materials Studio 8 (Biovia, San Diego, CA) and connected atoms were sequentially chosen until an approximately 1.6 nm particle was created (Fig. 1). Protons were added to the O atoms at the surface of the nanoparticle until the nanoparticle was charge neutral according to a scheme for predicting pK_a_s by Hiemstra [24]. The aid of Tjisse Hiemstra in this process was invaluable to derive a reasonably stable initial structure. We note that H^+^-transfers can readily occur even during 0 K energy minimizations during DFT calculations, so it is significant that no H^+^-transfers between model ferrihydrite nanoparticle surface sites were observed from the initial protonation states to the final minimum energy configurations. The only H^+^-transfer that occurred in this study was from a surface site to the ${\text{CrO}}_{4}^{2 - }$ ion to form ${\text{HCrO}}_{4}^{ - }$ during energy minimization of the outer-sphere species. The nanoparticle exhibits (001) and (100) surfaces but the surface Fe sites are predominantly associated with corners and edges of the nanoparticle. This small size and the predominance of surface defects likely increases the surface energy of the model compared to the larger observed ferrihydrite nanoparticles (2–10 nm), but practical computational constraints limit the size of the nanoparticle. A 2 nm particle was constructed, but the composition involved 96 Fe atoms, which rendered periodic DFT calculations impractical with the available computational resources. A ${\text{CrO}}_{4}^{2 - }$ ion was added to nanoparticle model in two monodentate, two bidentate binuclear, one outer-sphere, and one dissolved configuration. The four inner-sphere configurations were selected to test the thermodynamic favorability of surface site types (Fig. 2a–d).

**Fig. 3:**
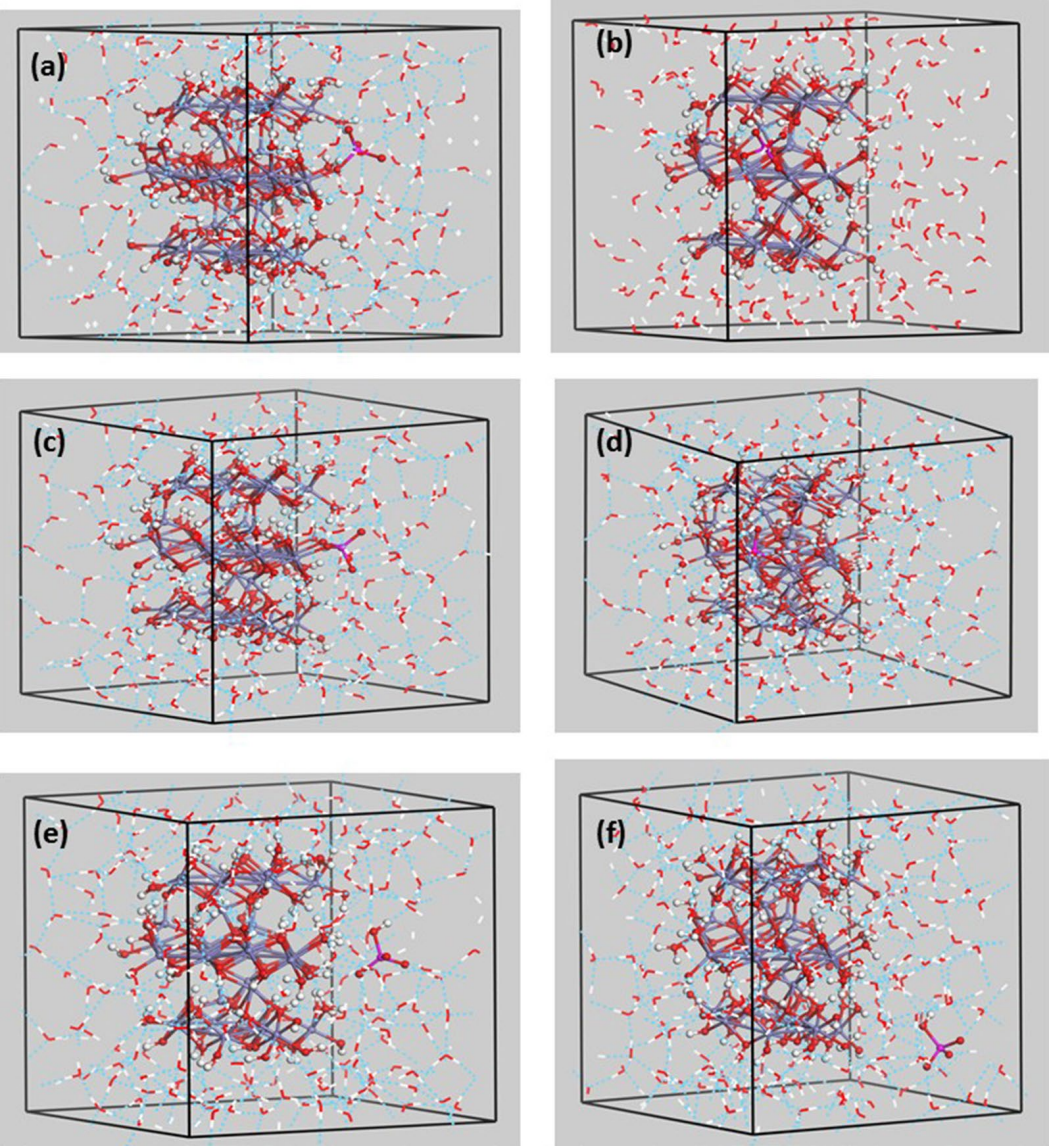
**a**–**f** The resulting structures from Fig. 2 were used as starting configurations for energy minimizations using periodic DFT methods for comparison with outer-sphere (**e**) and aqueous chromate (**f**)

The ferrihydrite + ${\text{CrO}}_{4}^{2 - }$ nanoparticle was centered in a 20 × 20 × 20 Å^3^ 3-D periodic box using the Crystal Builder module of Materials Studio 8 (Biovia, San Diego, CA). The volume of the nanoparticle and chromate was calculated using the Volume & Surface tool of Materials Studio 8, and this volume was subtracted from the volume of the periodic box in order to calculate the volume available for H_2_O molecules of solvation to be placed in the cell. 191 H_2_O molecules were added to the simulation cell using the solvation Impact module of Maestro 1 (Schrödinger Maestro, version 9.7, Schrödinger, LLC, New York, NY, 2014) H_2_O positions were energy-minimized within the Forcite module of Materials Studio 8 (Biovia, San Diego, CA) using the central valence force field (CVFF) [25] with the position of the Fe, Cr, O and H atoms of the ferrihydrite nanoparticle and chromate ions fixed. The resulting structures were then used as starting configurations for energy minimizations using periodic DFT methods (Fig. 3a–f).

### Computational methods

Periodic DFT calculations were performed with the Vienna Ab-initio Simulation Package 5.3.5 (VASP) [26–29]. Models were energy minimized using projector-augmented wave (PAW) pseudopotentials (Fe_pv, O, H and Cr_pv) in VASP 5.3.5 with GGA exchange correlation functional of Perdew, Burke, and Ernzerhof (PBE) [30, 31], a 500 eV energy cut-off and 1 k-point. The Fe spin configuration was taken from a model exhibiting the most stable spin ordering of the periodic ferrihydrite model [23]. The spin state of Cr was set to 0. The DFT + U approach was employed for Fe and Cr atoms [32], and a *U* parameter of 4 eV was used [33]. Self-consistent energy convergence (*EDIFF*) was set to 1 × 10^−4^ eV and the structural energy convergence criterion (*EDIFFG*) was set to − 0.02 eV/Å. Calculations were run on the Linux clusters run by The Pennsylvania State University Advanced Cyberinfrastructure Institute and on the Texas Advanced Computing Center (TACC) supercomputer LoneStar 5.

After energy minimization of the periodic models, molecular clusters were extracted representative of the monodentate, bidentate binuclear, and outer-sphere configurations by selecting the three Fe-octahedra and H-bonded H_2_O molecules nearby the chromate of interest. These clusters were energy minimized using Gaussian 09 [34] first with frozen Fe atoms to maintain an approximation of the surface structure then with all atoms relaxed. B3LYP/6-311+G(d,p) [35–40], M06-2X/6-311+G(d,p) [36, 40, 41], and PBE0/6-311+G(d,p) [36, 40, 42] exchange–correlation density functionals and basis sets were used to examine the potential effects of the methods on the results. After completing the energy minimization, frequency analyses were performed in Gaussian 09 and the resulting frequencies scaled by 0.967 and 0.947–0.952, and 0.991 respectively (for 6-311G(d,p)), based on the National Institute of Standards and Technology Computational Chemistry Comparison and Benchmark DataBase. Computed vibrational modes were visualized using Molden [43] to assess which IR-active modes were associated with chromate vibrations for comparison with observed IR frequencies. Comparision of the results obtained using the B3LYP, M06-2X, and PBE0 functionals, each coupled with the 6-311+G(d,p) basis set, showed that the B3LYP results correlated more closely with observation (Additional file 1: Table S1) than the results from the other methods did, so the B3LYP/6-311+G(d,p) values are reported in this paper.

### EXAFS analyses

EXAFS analysis was performed on a beamline X23A2 operated by the National Institute of Standards and Technology, at the National Synchrotron Light Source (Brookhaven National Laboratory, Upton, NY). Incident X-ray energy was scanned across the EXAFS region of the Cr K-edge (E = 5989 eV) using a Si(311) monochromator and a single-bounce harmonic rejection mirror. The monochromator was calibrated using Cr foil. Fluorescent X-rays were collected using a Stern-Heald fluorescence detector. Samples of chromate adsorbed on hematite were centrifuged and the resulting paste was spread evenly between two layers of Kapton tape that was mounted on the sample holder for analysis. Final spectra are the result of 5 averaged scans. An adsorption samples for EXAFS analysis was prepared in a nitrogen atmosphere and consisted of 5 g/L ferrihydrite and initial chromate concentration of 1 mM. Sample pH was adjusted to 6.0 by the dropwise addition of HCl. Ionic strength of 0.01 M NaCl was used.

Data were processed using the software suite Demeter [44]. Background subtraction was performed using Athena [44] and IFEFFIT [45] with a frequency cutoff parameter (Rbkg) set to 0.8. The Fermi energy (E0) was set to 6007 eV to produce the EXAFS spectra in terms of photoelectron wavenumbers (χ (k), k-weight = 3). The spectra were then converted to R-space by taking the Fourier transform of χ (k). Fitting was performed simultaneously on all datasets in R-space using Artemis [18] to determine the degeneracy (N), half-path length (R), and mean-square displacement (σ^2^) of the backscatterers, in the k range 3–12. The fitting model was identical to the model used for chromate adsorption on hematite [46], for spectra collected under the same conditions. This included single and double scattering paths for Cr → O→O, which were found to be significant contributors to the EXAFS signal.

### ATR FTIR analyses

Several ATR studies provide vibrational frequencies for chromate adsorbed on ferrihydrite under several experimental conditions [8, 19, 20]. In this study, ATR flow-through experiments were conducted at pH 7, in order to isolate the frequencies observed at neutral pH, which corresponds to the conditions simulated by the computational models. The ferrihydrite suspension used for the experiments was characterized previously [20], and had a specific surface area of 347 m^2^/g, with a particle size of 3 nm. The ATR–FTIR spectra were collected using a Bruker Alpha RT spectrometer with a diamond internal reflection element (IRE), operated by the OPUS V6 software. The FH film was prepared by depositing 25 μL of the suspension on the IRE and dried under an argon atmosphere. The flow cell was connected to a Metrohm USA 848 Titrino Plus titrator by a peristaltic pump with Tygon tubing, allowing for continuous pH adjustment, along with argon purging. Effluent pH was also measured to ensure equilibrium at pH 7.

The film was first flushed with 50 mL of the 50 mM NaCl solution at a flowrate 0.3 mL/min. Background spectra of the FH and HT films equilibrated with the electrolyte were collected at pH 7 prior to the adsorption experiments. The chromate solution concentration, 50 μΜ Cr in 49.95 mM NaCl was much lower than the aqueous detection limit of ATR-FTIR for chromate (10 mM), so that the observed signal was only a result of the surface species. Adsorption spectra were continuously collected and averaged for each 2 mL of the outflow solution, until 38 mL, when the signal reached equilibrium. All spectra were collected by averaging 600 scans at 4 cm^−1^ resolution, for wavenumbers between 4000 and 400 cm^−1^.

## Results and discussion

Energy minimizations using CVFF typically lowered the potential energy of the model systems on the order of 5 kJ/mol from the randomized structure of H_2_O molecules initially provided by Maestro. We note that CVFF tended to result in minimal H-bonding where most H–O distances between H_2_O molecules and between H_2_O molecules and the Fe-OH groups were greater than 2.5 Å. Energy minimizations with the DFT method described above could decrease the potential energy on the order of 1000 kJ suggesting that the H-bond network from CVFF was limiting the accuracy of the model structure. (Note that the ferrihydrite nanoparticle and chromate ion structures were previously approximated via DFT calculations, so this error could have been larger because 1000 kJ pertains predominantly to H-bonding and the H_2_O configuration only.) Although CVFF likely underestimates H-bonding, DFT methods such as those used here can overestimate H-bonding [47], so the reader is cautioned about the significant inaccuracies in the DFT results reported herein.

**Fig. 4:**
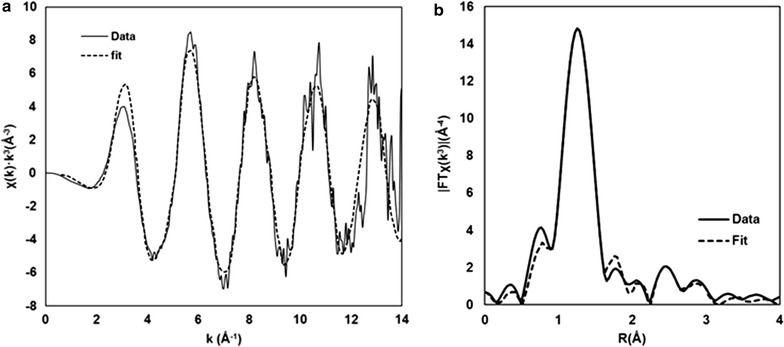
Cr K-edge EXAFS results for the data (solid lines) and model fit (dashed lines) for chromate adsorbed on ferrihydrite at pH 6: (**a**) k^3^-weighted χ (k) spectra and (β) Fourier transform magnitude

As a first test of the accuracy of the model results, comparisons to Cr–O bond lengths and Cr–Fe distances derived from EXAFS were made. The EXAFS results are shown in Fig. 4, Additional file 1: Table S1 and in the summary Table 1. Comparison in Table 1 reveals that all Cr–O distances in energy-minimized, 3-D periodic DFT models are within ± 0.02 Å of observed values. The model results are able to distinguish between the Cr–O bonds pointed away from the surface and the Cr–O(Fe) bonds as these are different by 0.04–0.05 Å. This is also true for the outer-sphere ${\text{HCrO}}_{4}^{ - }$ species which has three Cr–O bonds of ≈ 1.64 Å and a Cr–O(H) bond of 1.77 Å. Discerning these differences, rather than reporting a range of average values, would be useful in identifying monodentate versus bidentate surface complexes, so it would be worthwhile to perform EXAFS on chromate adsorbed to goethite and use these model results to help interpret the spectra. We caution that the Cr–O bond lengths could vary by as much as 0.08 Å depending on the H-bonding to the O atoms. Consequently, the hydration state of the samples in EXAFS experiments on adsorbed chromate is a significant factor in determining bond lengths. H-bonding networks for adsorbed oxyanions can be complex because the number and type of H-bonds are variable for each O atom within the oxyanion. O atoms may have 0–3 H-bonds and these H-bonds may come from H_2_O, or from surface OH or H_2_O groups. Consequently, determination of the H-bond state would best be determined via direct analytical methods or time averages from accurate molecular dynamics simulations.

**Table 1:** Calculated and observed Cr–O and Cr–Fe distances relevant to chromate adsorption onto Fe-oxides and Fe-hydroxides. Experiment data from other studies on goethite [22] and hematite [46] are shown

	Cr–O	Cr–O(Fe)	Cr–O(H)	Cr–Fe
Experiment (this study)	1.67			3.35–3.58
Experiment [22, 46]	1.64–1.68	–	–	3.30–3.60
Outer-sphere				
Periodic	1.64	–	1.77	5.27
M06-2x/6-31G(d)	1.57	–	1.80	4.17
M06-2x/6-311+G(d,p)	1.57	–	1.82	4.95
B3LYP/6-31G(d)	1.60	–	1.80	4.42
B3LYP/6-311+G(d,p)	1.60	–	1.83	4.54
PBE0/6-311+G(d,p)	1.59	–	1.81	5.80
Mono(A)				
Periodic	1.66	1.70	–	3.54
M06-2x/6-31G(d)	1.60	1.66	–	3.39
M06-2x/6-311 + G(d)	1.61	1.67	–	3.37
B3LYP/6-31G(d)	1.63	1.69	–	3.44
B3LYP/6-311+G(d,p)	1.63	1.69	–	3.40
PBE0/6-311+G(d,p)	1.61	1.68	–	3.35
Mono(B)				
Periodic	1.66	1.70	–	3.43
Bi(A)				
Periodic	1.65	1.70	–	3.29
M06-2x/6-31G(d)	1.57	1.68	–	3.19
M06-2x/6-311+G(d,p)	1.57	1.68	–	3.23
B3LYP/6-31G(d)	1.60	1.68	–	3.23
B3LYP/6-311+G(d,p)	1.60	1.69	–	3.26
PBE0/6-311+G(d,p)	1.59	1.68	–	3.22
Bi(B)				
Periodic	1.65	1.69	–	3.26
M06-2x/6-311+G(d,p)	1.59	1.66	–	3.40
B3LYP/6-311+G(d,p)	1.62	1.68	–	3.48
PBE0/6-311+G(d,p)	1.61	1.66	–	3.44

The Mono(B) configuration was not calculated as a cluster due to the prediction of a high total energy in the periodic model—see Table 2

The Cr–Fe distances do not distinguish well between the monodentate and bidentate models in comparison with the observed EXAFS (Table 1). In the periodic and cluster DFT calculations, the bidentate binuclear models fall within the lower end of the observed range (3.19–3.48 Å), and the Mono(A) configurations result in Cr–Fe distances at the higher end (3.35–3.54 Å) of the observed range. Thus, interpretation of EXAFS data should take into consideration that relaxation of monodentate surface complexes might result in similar metal–metal distances to those presumed for bidentate binuclear surface complexes [48]. Consideration of the metal–metal coordination number is critical in this case, but this parameter can often have significant uncertainty associated with it [49].

Table 2 contains relative energies of the six chromate-ferrihydrite models simulated with 3-D periodic DFT calculations. There are three key points to take away from these results. First, the periodic bidentate binuclear configuration B (Bi(B); Figs. 2d and 3d) is predicted to be the lowest in potential energy. This result is consistent with previous interpretations of chromate binding to Fe-oxides [22], and Fe-hydroxides [19], as mentioned in the section above. Second, the other three inner-sphere configurations are higher in energy than the outer-sphere configuration. The outer-sphere model is only +35 kJ/mol higher in energy than the lowest energy inner-sphere model (Bi(B)) which is not much larger than the expected computational error (± 10 kJ/mol) for our computational methodology on water–mineral oxide interfaces. When one considers that entropic factors are not included in the potential energy calculations, then the possibility that significant concentrations of the outer-sphere configuration may exist in equilibrium with the Bi(B) species must be considered. This situation is consistent with the observation for arsenate-hematite adsorption where similar amounts of inner- and outer-sphere species were observed via in situ resonant surface X-ray scattering measurements [21].

**Table 2:** Periodic (total energies, eV) and cluster (Gibbs free energies, Hartrees) with relative differences (ΔE and ΔG, respectively) among model configurations and the respective outer-sphere models which are set to 0 as a reference

	E	ΔE (kJ/mol)	G	ΔG (kJ/mol)
Aqueous	− 4309.16810	+ 60	–	–
Outer-sphere				
Periodic	− 4309.78815	0	–	–
M06/2x	− 6814.00142	0	− 6813.58455	0
B3LYP	− 6815.08577	0	− 6814.68635	0
PBE0	− 6811.91911	0	− 6811.50906	0
Mono(A)				
Periodic	− 4308.52000	+ 122	–	–
M06/2x	− 6813.98644	+ 39	− 6813.56964	+ 39
B3LYP	− 6815.07531	+ 27	− 6814.67559	+ 28
PBE0	− 6811.91929	− 1	− 6811.50402	+ 13
Mono(B)	− 4306.73599	+ 294	–	–
Bi(A)				
Periodic	− 4308.44527	+ 130	–	–
M06/2x	− 6813.97253	+ 76	− 6813.56314	+ 56
B3LYP	− 6815.07753	+ 22	− 6814.68274	+ 9
PBE0	− 6811.90971	+ 25	− 6811.50634	+ 7
Bi(B)				
Periodic	− 4310.15441	− 35	–	–
M06/2x	− 6813.96421	+ 98	− 6813.55141	+ 87
B3LYP	− 6815.07864	+ 19	− 6814.67830	+ 21
PBE0	− 6811.90861	+ 28	− 6811.49878	+ 27

The Mono(B) configuration was not calculated as a cluster due to the prediction of a high total energy in the periodic model

The third point taken from Table 2 is that the signs of the ΔG values calculated from the molecular clusters are generally (except for the PBE0 calculation on Mono(A)) the same sign as the ΔE values for the 3-D Mono(A) and Bi(A) periodic models. There are numerous differences between the ways the results were derived (i.e., computational methodology, long-range solid and solvent effects, inclusion of vibrational entropy, etc.), so one would not expect quantitative agreement in this case. The similar relative predicted thermodynamic stabilities in the periodic and cluster models suggest to a first approximation that short-range covalent bonding controls the stability of the surface complex. Factors such as pH and surface charge will be important as they affect the electrostatic component of the interaction energy, and these could be investigated by changing the H^+^/OH^−^ ratios in the models and assessing the effects on calculated ΔE and ΔG [16]. The lower absolute values of the cluster ΔG calculations are more consistent with measured ΔH values for oxyanions on Fe-oxy(hydr)oxide phases using flow adsorption calorimetry. Reported ΔH ranged in absolute values from ≈ 3.0–66 kJ/mol, with magnitudes increasing generally along a positive Hofmeister series [20, 50–52].

Unfortunately, the discrepancy in thermodynamic stability between periodic and cluster models arises for the Bi(B) configuration which was predicted to be most stable in the periodic model. Although one would expect the periodic models to better represent the actual adsorption chemistry, this discrepancy leads to some uncertainty in the prediction as to the most thermodynamically stable configuration.

**Fig. 5:**
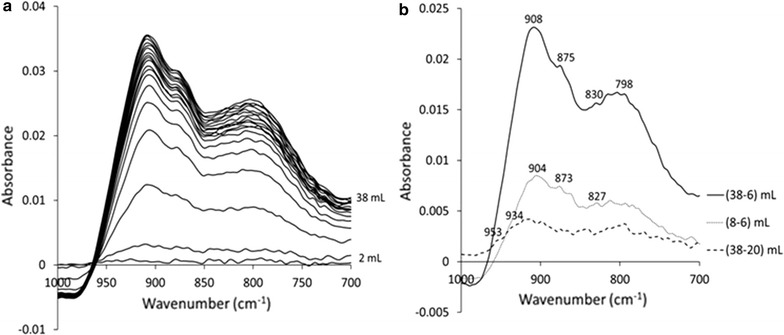
ATR FTIR spectra of chromate adsorbed on ferrihydrite at pH 7 (**a**) and difference spectra obtained at different surface coverages (**b**)

The ATR FTIR spectra shown in Fig. 5 are similar to spectra observed previously [19, 20], indicating that surface speciation at pH 7 is consistent with surface speciation observed over a range of pH values. The difference spectra show that the predominant species that is added both at low coverage (6–8 mL) and up to 20 mL has frequencies at 904–908, 873–875, 827–830 and 798 cm^−1^. These have been previously attributed to a monodentate species [8]. Higher frequencies are only observed at high coverage up to 38 mL and the difference spectra in this case have a low signal-to-noise ratio. Two additional peaks at 953 and 934 cm^−1^ may be discerned, which are consistent with the bidentate frequencies reported previously. This analysis indicates that at pH 7, the monodentate species is dominant, with some bidentate binuclear species also forming at high coverage.

**Fig. 6:**
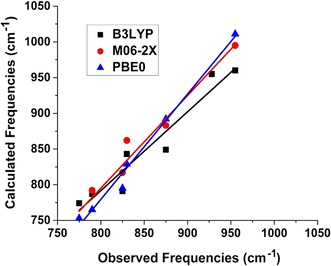
Correlations of calculated versus vibrational frequencies for bidentate binuclear models with the B3LYP, M06-2X and PBE0 exchange–correlation functionals and the 6-311+G(d,p) basis set. Correlation parameters listed in Additional file 1: Table S1

**Fig. 7:**
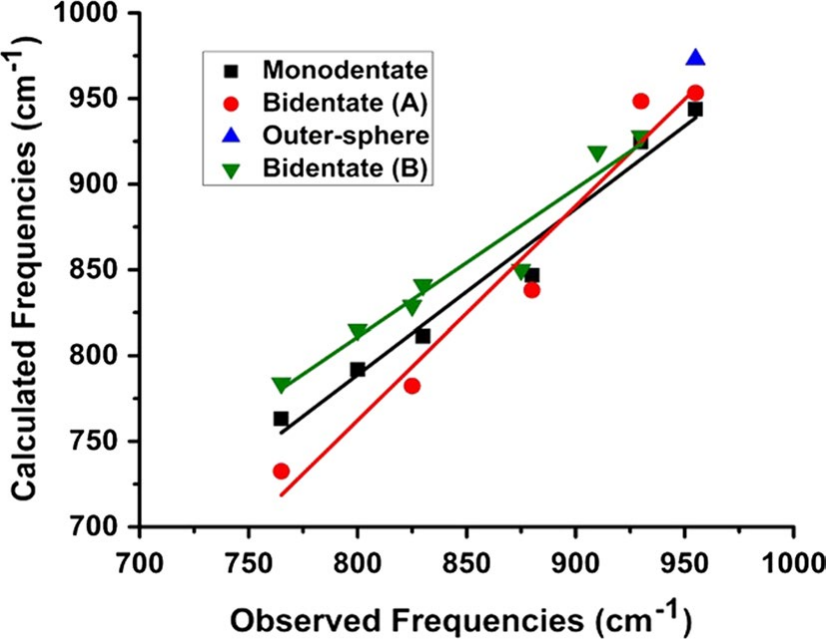
Correlations of calculated versus vibrational frequencies for monodentate, outer-sphere, and bidentate, binuclear (A and B) models with the B3LYP/6-311+G(d,p) basis set. Correlation parameters listed in Additional file 1: Table S1

Only the frequencies from the B3LYP/6-311+G(d,p) calculations are listed in Table 3 because this method provides better overall correlations with observed frequencies compared with M06-2× and PBE0 in this case (Fig. 6 and Additional file 1: Table S1) with the notable exception of the Bi(B) model with M06-2x that has excellent agreement with experiment (Additional file 1: Table S1). Examination of Table 3 reveals two important conclusions. First, all of the observed IR frequencies can be explained by the model results. Second, under a given set of experimental conditions, one surface complex cannot explain all of the observed frequencies. These results are consistent with the energy calculations that predict bidentate binuclear and outer-sphere complexes may exist simultaneously in finite concentrations (Table 2). The monodentate species was not predicted to have any significant stability, however, several vibrational modes of the monodentate model correspond to observed IR frequencies (Fig. 7, Table 3). In fact, the monodentate model results in a better correlation with observed frequencies than the bidentate (B) model using B3LYP (Additional file 1: Table S1), but the monodentate model does not produce a peaks in neither the 820–825 nor 905–910 cm^−1^ range to match observation (Table 3). The assignment of particular IR peaks to monodentate chromate may be complicated by the fact that the calculated outer-sphere and monodentate models have some similar frequencies (Table 3). It is also likely that surface coverage effects decrease the number of bidentate sites that can be formed, so monodentate complexes form in their place. Future modeling involving competitive effects of adsorbing multiple chromate molecules would be necessary to address this question.

**Table 3:** Observed and scaled calculated IR-active frequencies (cm^−1^) for chromate adsorbed onto ferrihydrite

Observed/assignment	Outer-sphere	Mono(A)	Bi(A)	Bi(B)
765/Bi	–	763	732	784
800/Mono^a^	–	792	782	815
	–	811	–	–
820-825/Mono^b^	–	–	–	829
830/OS + Bi^a^	856	846	838	841
873-875/OS + Mono	874	874	–	850
880/Bi	–	–	–	–
905-910/Mono	–	924	–	919
930/Bi	–	944	948	928
955/Bi	973	–	953	–
	1003	–	962	–
	1022	–	–	–
	1063	–	–	–
	1070	–	–	–
	1083	–	–	–
	1100	–	–	–

B3LYP/6-311+G(d,p) scaled by 0.967 (NIST Computational Chemistry Comparison and Benchmark DataBase)

^a^Ref. [19]

^b^Ref. [8, 20], and this study

A secondary issue is that model results predict frequencies above 1000 cm^−1^ that were not detected in the ATR FTIR spectra. A similar phenomenon has occurred before modeling phosphate adsorption onto goethite where higher-frequency peaks associated with P-OH vibrational modes were not detected in observed spectra. This discrepancy could be an issue with the model constructed, the computational methodology, and/or detection problems in the spectra collection. Most vibrational modes are modeled reasonably accurately with the DFT methods applied here as demonstrated by comparisons of modeled and observed frequencies on aqueous species [10]. However, modeling some modes is problematic, notably those involving metal–oxygen double bonds where electron correlation is significantly more important [53]. These stronger bonds are likely to have higher frequencies compared to single bonds, so this limitation of the applied DFT techniques is likely a source of error here. In addition, the observed peaks are broad and background subtraction can be problematic, so it is possible that some IR peaks are not detected in the observed spectra as well.

## Summary

Although the complexity of the system and limitations of the computational methodology and models employed lead to ambiguity on some questions, the following conclusions can be made:

1. The bidentate bridging complex is most likely to give rise to the EXAFS and predominant IR spectral features consistent with previous interpretations that it is forms the highest concentration of surface complexes of chromate on ferrihydrite.
2. Under most circumstances, an equilibrium among outer-sphere and inner-sphere complexes is likely to exist. The relative ratio of the various complexes will be a function of numerous parameters (e.g., pH, ionic strength, etc.) that are beyond the scope of this study.
3. Changing surface concentrations of chromate will shift the ratios of bidentate and monodentate complexes as these shifts are observed via detection of specific peaks in the IR spectra.
4. DFT results clearly indicate a significant degree of variability in adsorption energies (ΔE_ads_) at various sites so this variability be included in future DFT studies attempting to compare to adsorption calorimetry data.
5. Energy minimizations based on CVFF-derived nanoparticle-water structures are inadequate predicting adsorption thermodynamics. DFT-MD simulations and inclusion of pH and ionic strength effects may improve the accuracy of DFT-produced thermodynamic predictions.

## Additional file

**Additional file 1: Table S1.** Cr K-edge fitting results for the local structure of chromate adsorbed to ferrihydrite based on the monodentate and bidentate two-complex model. The fitting was evaluated with a reduced χ2 = 7.32 and a goodness of fit (R-factor) of 0.009. **Table S2.** Frequency correlations with various exchange correlation functionals using the 6-311+G(d,p) basis set. Note values are not listed for the outer-sphere models because calculated frequencies only match with one observed frequency near 955 cm^−1^.

## Abbreviations

ATR FTIR: Attenuated Total Reflectance Fourier-Transform Infrared
DFT: density functional theory
EXAFS: extended X-ray absorption fine structure
