# Supplementary material for: Density functional theory modeling of chromate adsorption onto ferrihydrite nanoparticles
Source: Geochem Trans. 2018 Mar 1;19:8. doi: 10.1186/s12932-018-0053-8 (PMC5832661; doi:10.1186/s12932-018-0053-8)
Supplement: Supplementary file 1 — Additional file 1: Table S1. Cr K-edge fitting results for the local structure of chromate adsorbed to ferrihydrite based on the monodentate and bidentate two-complex model. The fitting was evaluated with a reduced χ2 = 7.32 and a goodness of fit (R-factor) of 0.009. Table S2. Frequency correlations with various exchange correlation functionals using the 6-311+G(d,p) basis set. Note values are not listed for the outer-sphere models because calculated frequencies only match with one observed frequency near 955 cm−1. [file 12932_2018_53_MOESM1_ESM.docx]

**Additional Information**

**Additional file 1: Table S1 -** Cr K-edge fitting results for the local structure of chromate adsorbed to ferrihydrite based on the monodentate and bidentate two-complex model. The fitting was evaluated with a reduced χ2 = 7.32 and a goodness of fit (R-factor) of 0.009.

| **Path** | **N^a^** | **R (Å)** | **σ^2^ (Å^2^)^b^** |
| --- | --- | --- | --- |
| Cr→O | 4.0 | 1.65 | 0.0014 |
| Cr→O→O | 12.0 | 3.00 | 0.0014 |
| Cr→Fe | 0.7 | 3.35 | 0.0107 |
| Cr→Fe | 0.4 | 3.58 | 0.0107 |

^a^ N values for Cr-O single and multiple scattering paths were fixed to theoretical values.

^b^ Global value floated for each Fe path.

**Additional file 1: Table S2** - Frequency correlations with various exchange correlation functionals using the 6-311+G(d,p) basis set. Note values are not listed for the outer-sphere models because calculated frequencies only match with one observed frequency near 955 cm^-1^.

**Slope Intercept R^2^ Residual Sum of Squares**

**Bidentate binuclear**

**B3LYP** 1.25±0.14 -237±125 0.95 1473

**M06-2X** 1.30±0.12 -243±106 0.95 1359

**PBE0** 1.47±0.08 -400±71 0.98 604

**Bidentate binuclear (B)**

**B3LYP** 0.87±0.09 118±79 0.94 912

**M06-2X** 0.99±0.04 18±37 0.99 136

**PBE0** 0.81±0.18 163±157 0.86 1007

**Monodentate**

**B3LYP** 0.97±0.07 15±65 0.97 624

**M06-2X** 1.27±0.21 -227±188 0.85 4398

**PBE0** 0.94±0.08 48±66 0.97 397
